# MAP Tag: A Novel Tagging System for Protein Purification and Detection

**DOI:** 10.1089/mab.2016.0039

**Published:** 2016-12-01

**Authors:** Yuki Fujii, Mika K. Kaneko, Yukinari Kato

**Affiliations:** Department of Regional Innovation, Tohoku University Graduate School of Medicine, Miyagi, Japan.

**Keywords:** monoclonal antibody, MAP tag, affinity tag, protein purification

## Abstract

Protein purification is an essential procedure in fields such as biochemistry, molecular biology, and biophysics. Acquiring target proteins with high quality and purity is still difficult, although several tag systems have been established for protein purification. Affinity tag systems are excellent because they possess high affinity and specificity for acquiring the target proteins. Nevertheless, further affinity tag systems are needed to compensate for several disadvantages of the presently available affinity tag systems. Herein, we developed a novel affinity tag system designated as the MAP tag system. This system is composed of a rat anti-mouse podoplanin monoclonal antibody (clone PMab-1) and MAP tag (GDGMVPPGIEDK) derived from the platelet aggregation-stimulating domain of mouse podoplanin. PMab-1 possesses high affinity and specificity for the MAP tag, and the PMab-1/MAP tag complex dissociates in the presence of the epitope peptide, indicating that the MAP tag system is suitable for protein purification. We successfully purified several proteins, including a nuclear protein, soluble proteins, and a membrane protein using the MAP tag system. The MAP tag system is very useful not only for protein purification but also in protein detection systems such as western blot and flow cytometric analyses. Taken together, these findings indicate that the MAP tag system could be a powerful tool for protein purification and detection.

## Introduction

Affinity tag systems are highly useful for protein purification, detection, and analysis. Affinity tag systems are classified broadly as “peptide tags” and “protein tags.” For example, GST tag^(1)^ and MBP tag^(2)^ are protein tags. While these tags are useful for soluble fraction proteins, they are large (over 25 kDa) and thus can affect target protein function, requiring their removal before protein analysis. In contrast, peptide tags are less likely to affect the structure and function of target proteins because they are small (typically 1–2 kDa). Thus, removal of the tag portion is not always required before protein analysis. The most appropriate peptide tag system can be chosen from a variety of options, including the His tag,^(3)^ FLAG tag,^(4)^ TARGET tag,^(5)^ PA tag,^(6,7)^ AGIA tag,^(8)^ and many others.^(9,10)^ The fusion peptides are compatible with virtually all expression host cells, including yeast, insect, and mammalian cells, and *Escherichia coli* and can be expressed extracellularly or intracellularly.

An excellent affinity tag system should have high affinity and high specificity. However, not all peptide-based tag systems meet these criteria. For example, the purification of oligohistidine-tagged proteins using metal chelate affinity resin often results in the co-purification of metal-binding proteins present in the starting material, necessitating further purification steps.^(11)^ Generally, epitope tag systems that utilize peptide tags and anti-peptide monoclonal antibodies (mAbs) are highly specific. However, we often encounter nonspecific binding of mAbs to endogenous proteins in certain cell types,^(12,13)^ even when using the most popular tag system, FLAG tag/anti-FLAG M2 antibody.^(14)^ Importantly, the most suitable tag systems must be chosen based on the target protein, expression host, and many other variables. Therefore, the development of further affinity tag systems is needed to overcome the disadvantages of available affinity tag systems.

We previously established a useful rat mAb (clone PMab-1) against a 14-residue peptide in the platelet aggregation-stimulating (PLAG) domain of mouse podoplanin.^(15)^ Podoplanin is a type I transmembrane protein that is highly expressed in malignant cancer cells and is implicated in tumor-induced platelet aggregation.^(16)^ In our another study, we developed the PA tag system with high affinity and specificity.^(6)^ The PA tag is derived from the human podoplanin PLAG domain. Three tandem repeats of the PLAG domain are conserved in podoplanin orthologs of the rat, hamster, dog, cow, human, and mouse.^(17)^ Additionally, PMab-1 possesses high affinity and specificity toward mouse podoplanin.^(18)^ Therefore, it was predicted that PMab-1 would have characteristics suitable for an anti-tag antibody. Here, we report the development of a novel affinity tag system comprising PMab-1 and its epitope peptide MAP tag.

## Materials and Methods

### Cell lines

LN229, HEK293T, COS-7, and Chinese hamster ovary (CHO)-K1 cell lines were purchased from the American Type Culture Collection (ATCC, Manassas, VA). LN229 was transfected with epidermal growth factor receptor (EGFR), the entire ectodomain of human EGFR (EGFR_ec_), the entire ectodomain of human HER2 (HER2_ec_), and CD133 plasmids (LN229/EGFR, LN229/EGFR_ec_, LN229/HER2_ec_, and LN229/CD133, respectively) using a Neon transfection system (Thermo Fisher Scientific, Inc., Waltham, MA). CHO-K1 was transfected with CD133 plasmid (CHO/CD133) using Lipofectamine LTX (Thermo Fisher Scientific, Inc.). HEK293T, COS-7, and CHO-K1 cells were transiently transfected with the hPDPNdN55 plasmid (HEK293T/hPDPNdN55, COS-7/hPDPNdN55, and CHO/hPDPNdN55, respectively) using Lipofectamine LTX. LN229, HEK293T, COS-7, LN229/EGFR, LN229/EGFR_ec_, LN229/HER2_ec_, and LN229/CD133 cells were cultured in Dulbecco's modified Eagle's medium including 2 mM l-glutamine (Nacalai Tesque, Inc., Kyoto, Japan). CHO-K1 and CHO/CD133 were cultured in RPMI 1640 medium including 2 mM l-glutamine (Nacalai Tesque, Inc.), supplemented with 10% heat-inactivated fetal bovine serum (Thermo Fisher Scientific, Inc.), 100 U/mL penicillin, 100 mg/mL streptomycin, and 25 mg/mL amphotericin B (Nacalai Tesque, Inc.) at 37°C in a humidified atmosphere of 5% CO_2_.

### Plasmid preparation

Human ATRX cDNA encoding amino acids 2273–2413 was obtained by polymerase chain reaction (PCR) using a cDNA derived from human lung as a template.^(19)^ DNA encoding the PA tag (GVAMPGAEDDVV), RAP tag (DMVNPGLEDRIE), and MAP tag (GDGMVPPGIEDK) was inserted into the NdeI-XhoI site of pET21b vector (Novagen; EMD Millipore Corp., Billerica, MA) using the In-Fusion PCR cloning kit (Takara Bio, Inc., Shiga, Japan) (PA-RAP-MAP/pET21b vector). The expression construct for recombinant ATRX (amino acids 2273–2413) was cloned into the EcoRI site of PA-RAP-MAP/pET21b vector (PA-ATRXepi-RAP-MAP/pET21b). The DNAs encoding human EGFR, human HER2, and human CD133 were obtained by PCR using cDNAs derived from A431, A172, and HCT116 as a template, respectively. RAP tag and MAP tag were inserted into the BamHI-NotI sites of pCAG PA tag-N vector (Wako Pure Chemical Industries Ltd., Osaka, Japan) using the In-Fusion PCR cloning kit (PA-RAP-MAP/pCAG vector). DNAs encoding EGFR, EGFR_ec_, HER2_ec_, and CD133 were cloned into PA-RAP-MAP/pCAG vector (PA-EGFR-RAP-MAP/pCAG, PA-EGFR_ec_-RAP-MAP/pCAG, PA-HER2_ec_-RAP-MAP/pCAG, and PA-CD133-RAP-MAP/pCAG, respectively). The MAP-tagged deletion mutant of human podoplanin without the PLAG domain (MAP-hPDPNdN55/pCAG) was cloned into the pCAG vector as described previously.^(20)^ All constructs were verified by DNA sequencing.

### Binding kinetics analysis using surface plasmon resonance

Rat anti-MAP mAb PMab-1 (IgG_2a_, κ) was described previously.^(15)^ PMab-1 was immobilized on CM5 sensor chips using amino coupling chemistry according to the method provided by the manufacturer. The MAP-tagged ATRXepi proteins were diluted in PBST (phosphate-buffered saline, pH 7.4, containing 0.005% Tween 20) and injected at a flow rate of 30 μL/min. The ATRXepi was diluted in PBST (12.5, 25, 50, 100, and 200 nM) and passed over the biosensor chip. The binding was monitored for 60 seconds, followed by dissociation in running buffer for 120 seconds using the Single-cycle kinetics method. Binding curves were analyzed using BIAcore X100 Evaluation Software (GE Healthcare, Piscataway, NJ) with curve fitting using a 1:1 binding model.

### Western blot analyses

Cell lysates (10 μg) were boiled in sodium dodecyl sulfate (SDS) sample buffer (Nacalai Tesque, Inc.). The proteins were electrophoresed on 5%–20% polyacrylamide gels (Wako Pure Chemical Industries Ltd.) and transferred onto a PVDF membrane (EMD Millipore Corp.). After blocking with 4% skim milk (Nacalai Tesque, Inc.), the membrane was first incubated with 1 μg/mL PMab-1, anti-β-actin (AC-15; Sigma-Aldrich, St. Louis, MO), anti-IDH1 (RcMab-1),^(21)^ or an anti-RAP tag (PMab-2), and then with 1:1000 diluted peroxidase-conjugated anti-rat or mouse IgG (Dako; Agilent Technologies, Inc., Glostrup, Denmark), followed by development in Pierce Western Blotting Substrate Plus (Thermo Fisher Scientific, Inc.) using a Sayaca Imager (DRC Co. Ltd., Tokyo, Japan).

### Flow cytometry

The mouse anti-human podoplanin mAb LpMab-17 (IgG_1_, κ) was described previously.^(22)^ Cell lines were harvested by brief exposure to 0.25% Trypsin/1 mM EDTA (Nacalai Tesque, Inc.). After washing with PBS, the cells were treated with PMab-1 (1 μg/mL) or LpMab-17 (1 μg/mL) for 30 minutes at 4°C, followed by treatment with 1:1000 diluted Oregon Green 488 goat anti-rat or mouse IgG (Thermo Fisher Scientific, Inc.). Fluorescence data were collected using a Cell Analyzer EC800 (Sony Corp., Tokyo, Japan).

### Protein expression and purification

PMab-1 was coupled to CNBr-activated Sepharose 4B (GE Healthcare) according to the protocol provided by the manufacturer, routinely yielding coupling levels of ∼2 mg IgG/mL gel. Recombinant ATRXepi (PA-ATRXepi-RAP-MAP) was expressed in transformed *E. coli* BL21 (DE3) cultured for ∼3 hours at 37°C in the presence of 1 mM IPTG, and the bacterial pellet from a 100 mL culture was suspended in 10 mL of Tris-buffered saline (TBS; pH 7.5). Bacteria were lysed by sonication, and the soluble fraction was obtained by centrifugation at 15,000 *g* for 15 minutes. The lysates were passed through a 0.45 μm filter to remove any trace amounts of insoluble materials. The cleared lysates were mixed with PMab-1-Sepharose (1 mL bed volume) and incubated at 4°C for 3 hours under gentle agitation. The beads were then transferred to a column and washed with 20 mL of TBS. The bound protein was eluted with TBS containing 0.1 mg/mL epitope peptide (GDGMVPPGIEDKIT). The elution was conducted at room temperature in a stepwise manner (1 mL × 10), with a dissociation time of 5 minutes allowed for each elution step.

For the purification of soluble EGFR_ec_ and HER2_ec_ secreted from mammalian cells, LN229/EGFR_ec_, and LN229/HER2_ec_ were plated in 225 cm^2^ flask (Thermo Fisher Scientific, Inc.), and 1 L of culture supernatant was harvested. Cleared supernatant was passed through PMab-1-Sepharose (4 mL bed volume), and the same process was repeated three times. The beads were then washed with 80 mL of TBS and eluted with the same solution containing 0.1 mg/mL epitope peptide in a stepwise manner (4 mL × 10) as described above. For the purification of EGFR from mammalian cell membranes, LN229/EGFR was plated onto hundred 10-cm culture dishes. Cells were detached from the plates using PBS containing 1 mM EDTA. The pelleted cells were washed using PBS and solubilized by adding 10 mL of PBS containing 1% (w/v) Triton X-100 and 0.05 mg/mL aprotinin. The solubilized cell lysates were incubated at 4°C for 30 minutes and further centrifuged 15 minutes at 13,000 *g* at 4°C. The pelleted cells were solubilized in 5 mL of the same solution, and the process was repeated twice. The cleared lysates were mixed with PMab-1-Sepharose (1 mL bed volume) and incubated at 4°C for 3 hours under gentle agitation. The beads were then transferred to a column and washed with 20 mL of PBS. The bound protein was eluted with PBS containing 0.1 mg/mL epitope peptide in a stepwise manner (1 mL × 10) as described above.

Ten peptide-elution fractions from the column chromatography were subjected to 5%–20% SDS-PAGE under reducing conditions and stained with Coomassie Brilliant Blue (CBB) or Oriole fluorescent protein stain (Bio-Rad Laboratories Inc., Berkeley, CA).

## Results

### Kinetic analysis of PMab-1/MAP tag interaction

**FIG. 1.**
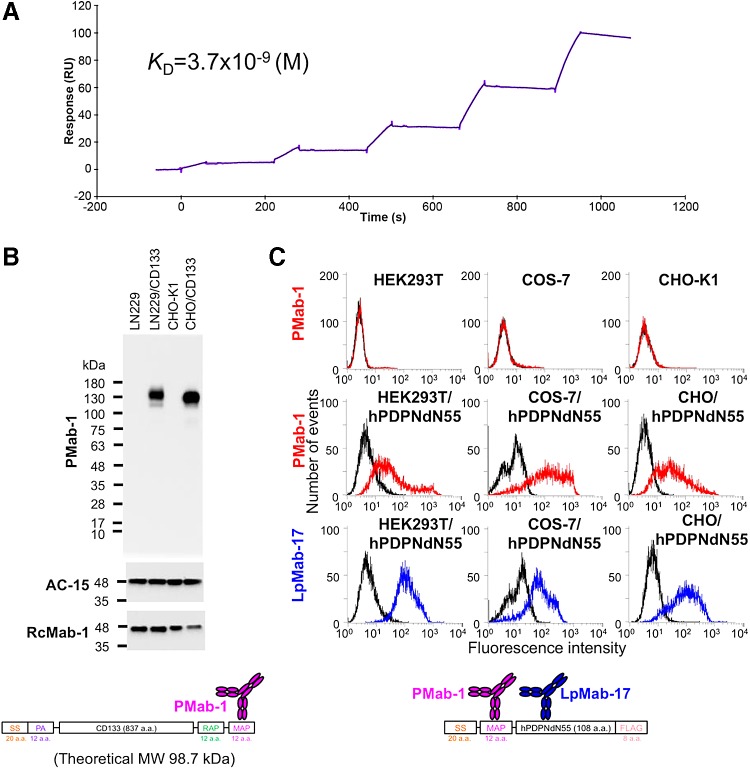
Characterization of the MAP tag system. **(A)** Surface plasmon resonance kinetic analysis of PMab-1 binding toward the MAP tag. Serially diluted, MAP-tagged ATRXepi (12.5, 25, 50, 100, and 200 nM) was injected over the CM5 sensor chip immobilized with PMab-1 for 60 seconds, followed by dissociation in PBST for 120 seconds at a flow rate of 30 μL/min. The binding curve was globally fitted to a 1:1 binding model to derive the equilibrium dissociation constant (*K*_D_) values shown. **(B)** Western blot analysis of MAP tagged CD133 with PMab-1. Total cell lysates (LN229, LN229/CD133, CHO-K1, and CHO/CD133) were electrophoresed under reducing conditions on a 5%–20% SDS-PAGE gel and transferred to a membrane. A membrane containing the same amount of lysate was immunoblotted with 1 μg/mL PMab-1, AC-15, or RcMab-1 for 30 minutes and incubated with peroxidase-conjugated secondary antibody specific for rat (PMab-1 and RcMab-1) or mouse (AC-15) IgG. **(C)** Flow cytometric analysis of MAP-tagged membrane protein. HEK293T, COS-7, and CHO-K1 cells and those transiently transfected with MAP-hPDPNdN55 were incubated with 1 μg/mL PMab-1 or LpMab-17 and stained with Oregon Green 488-labeled secondary antibody specific for rat or mouse IgG. CHO, Chinese hamster ovary; SDS-PAGE, sodium dodecyl sulfate polyacrylamide gel electrophoresis; SS, signal sequence.

First, we investigated whether PMab-1 maintains a high affinity toward MAP tag “GDGMVPPGIEDK.” We analyzed the binding affinity between PMab-1 and MAP tag using the BIAcore X100 system. Curve-fitting analysis showed high-affinity PMab-1/MAP tag interaction (*k_a_*, 1.1 × 10^5^ M^−1^s^−1^; *k_d_*, 4.2 × 10^−4^ s^−1^; *K*_D_, 3.7 × 10^−9^ M) (Fig. 1A).

### Specificity analysis of PMab-1/MAP tag interaction

We next evaluated the specificity of PMab-1 for the MAP tag. As shown in Figure 1B, PMab-1 produced a strong single band corresponding to the expressed CD133 with MAP tag (∼120 kDa) in western blot analysis of the cell lysate. PMab-1 did not show any reactive bands in CHO-K1 cells, indicating that PMab-1 is very specific against the MAP tag. The specificity was also analyzed using flow cytometry (Fig. 1C). PMab-1 reacted with the hPDPNdN55 plus N-terminal MAP tag (extracellular domain), which is expressed in HEK293T, COS-7, and CHO-K1 cells (Fig. 1C, middle panel) and did not react with the corresponding untransfected cells (Fig. 1C, upper panel). LpMab-17, a mouse anti-human podoplanin mAb, recognized all transfected cell lines (Fig. 1C, lower panel).

### Application to the protein purification system

**FIG. 2.**
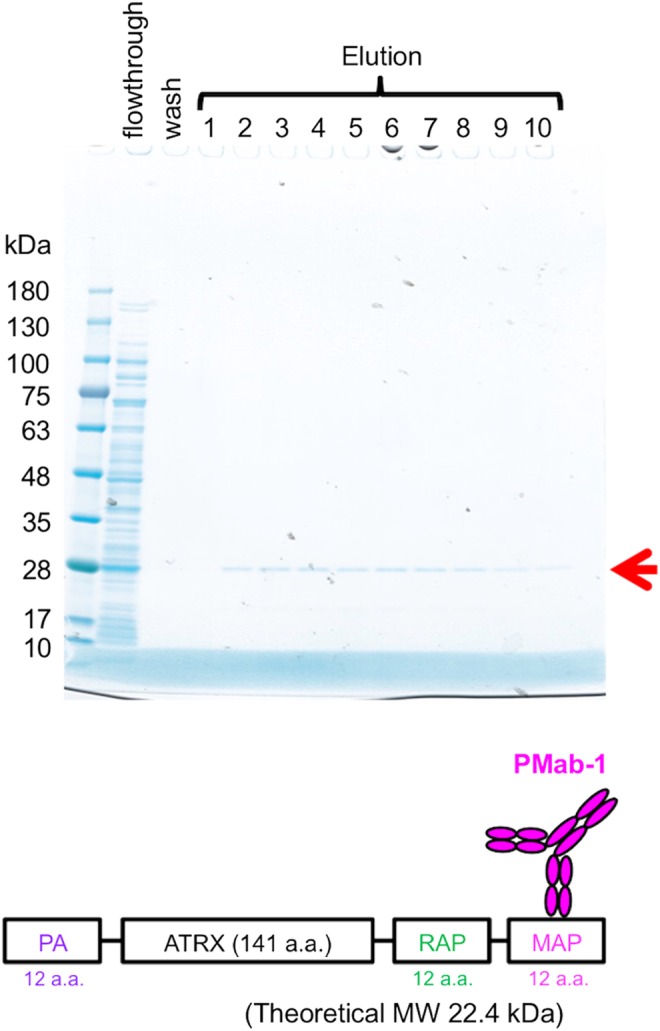
Purification of the ATRX fragment (ATRXepi) from transformed *E. coli*. The soluble fraction (5 μL) from the transformed *E. coli* after PMab-1-Sepharose capture (flowthrough), and the fifth of five washes in 20 mL TBS (wash), and 10 peptide-elution fractions (lanes 1–10) from the column chromatography were subjected to 5%–20% SDS-PAGE under reducing conditions and stained with CBB. TBS, Tris-buffered saline; CBB, Coomassie Brilliant Blue.

**FIG. 3.**
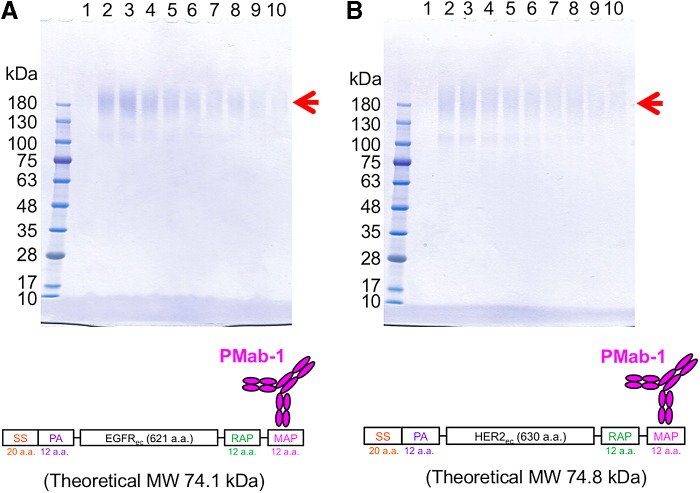
Purification of the soluble ectodomain fragments of EGFR_ec_ **(A)** and HER2_ec_ **(B)**. PMab-1-Sepharose captured these proteins in the culture supernatant from transfected LN229 and was washed with 20 mL TBS. Ten peptide-elution fractions (10 μL) (lanes 1–10) from the column chromatography were subjected to 5%–20% SDS-PAGE under reducing conditions and stained with CBB. SS, signal sequence; EGFR, epidermal growth factor receptor; CBB, Coomassie Brilliant Blue.

**FIG. 4.**
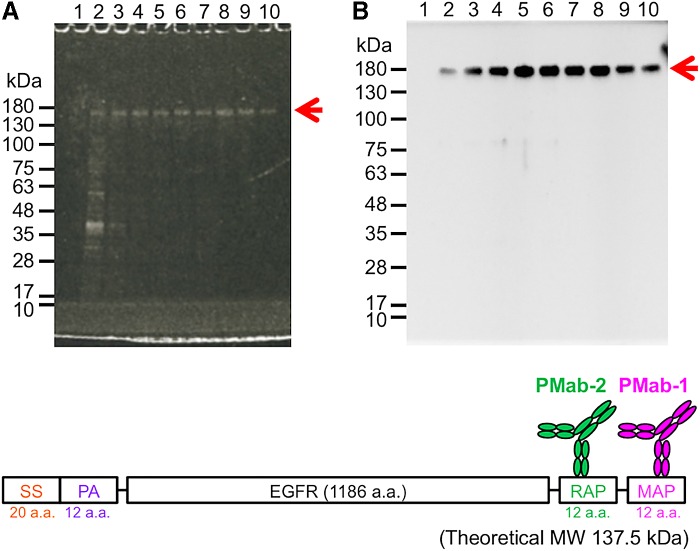
Purification of EGFR from LN229 cells. PMab-1-Sepharose captured the proteins in the detergent-solubilized cell lysates from transfected LN229 and was washed with 20 mL PBS. **(A)** Ten peptide-elution fractions (10 μL) (lanes 1–10) from the column chromatography were subjected to 5%–20% SDS-PAGE under reducing conditions and stained with Oriole fluorescent protein stain. **(B)** Ten peptide-elution fractions (5 μL) (lanes 1–10) from the column chromatography were subjected to 5%–20% SDS-PAGE under reducing conditions and transferred to a membrane. The membrane was immunoblotted with 1 μg/mL of PMab-2 (anti-RAP tag) for 30 minutes and incubated with peroxidase-conjugated secondary antibody specific for mouse IgG. PBS, phosphate-buffered saline; SS, signal sequence.

We attempted to purify the four proteins with MAP tag using the MAP tag system. ATRXepi (∼25 kDa) was expressed in *E. coli* and purified from the soluble fraction of the bacterial lysate (Fig. 2). This protein eluted in fractions 2–10, with virtually no contamination. The soluble ectodomain fragments of EGFR (EGFR_ec_) and HER2 (HER2_ec_) were expressed in LN229 and purified from the culture supernatant (Figs. 3A and 3B). Because EGFR_ec_ and HER2_ec_ are highly glycosylated, they electrophoresed more slowly than predicted by the theoretical molecular weights. These proteins were eluted in elution fractions 2–10, with virtually no contamination. EGFR (∼170 kDa) was expressed in LN229 and was purified from the membrane fraction of LN229 (Fig. 4). This protein eluted in fractions 2–10, and the single bands were detected in elution fractions 4–10 (Fig. 4A). It was also confirmed that the strong bands in elution fractions were derived from EGFR in western blot analysis using an anti-RAP tag mAb (clone: PMab-2) (Fig. 4B). All MAP-tagged proteins were captured onto PMab-1-Sepharose and eluted from the resin with a solution containing 0.1 mg/mL free epitope peptide (GDGMVPPGIEDKIT). Despite the high affinity between PMab-1 and MAP tag (Fig. 1A), these antigens were eluted by the peptide. We performed the elution at room temperature in a stepwise manner, with a dissociation time of 5 minutes allowed for each elution step. These eluted proteins showed high purity (Figs. 2–4).

## Discussion

An excellent affinity tag system requires high affinity and specificity. PMab-1, a rat anti-mouse podoplanin mAb, was produced by immunization with the synthetic peptide “GDGMVPPGIEDKIT”.^(15)^ PMab-1 has high affinity and specificity toward mouse podoplanin as described previously.^(18)^ Therefore, we investigated whether PMab-1 maintains high affinity and specificity toward the MAP tag “GDGMVPPGIEDK” that was attached to several proteins.

First, we analyzed the binding affinity between PMab-1 and the MAP tag. Curve-fitting analysis showed high-affinity PMab-1/MAP tag interaction (*K*_D_, 3.7 × 10^−9^ M) (Fig. 1A). These data do not differ from those of previous studies that analyzed PMab-1/mouse podoplanin interaction using flow cytometry (*K*_D_, 3.2 × 10^−9^ M).^(18)^ Next, we evaluated the specificity of PMab-1 against MAP tag. As shown in Figures 1B and 1C, PMab-1 has high specificity toward MAP tag in Western blot analysis and flow cytometry. Although HEK293T cells are reported to express human podoplanin,^(23)^ PMab-1 did not react with untransfected HEK293T cells because PMab-1 does not cross-react with human podoplanin. In fact, the important amino acids for PMab-1/MAP tag interaction are the first aspartic acid and the methionine in the MAP tag (data not shown); human podoplanin does not have this sequence. These results indicate that PMab-1 has a high affinity and specificity toward not only mouse podoplanin but also the MAP tag. Therefore, we presumed that the PMab-1/MAP tag system would be applicable to the protein purification system.

We attempted to purify MAP-tagged ATRXepi, EGFR_ec_, HER2_ec_, and EGFR using the MAP tag system (Figs. 2–4). These purified proteins showed high purity and were ready for use in the downstream experiments without further purification. Purifying membrane proteins is generally difficult because membrane proteins require detergent to make them soluble in an aqueous solution. In contrast, the MAP tag system purified membrane proteins such as EGFR in the presence of detergent (Fig. 4). These results indicate that the MAP tag system is a powerful protein purification tool that does not depend on the host or the type of protein.

In conclusion, we successfully developed a novel affinity tagging system designated “MAP tag” that employs a unique mAb PMab-1 against mouse podoplanin. Owing to its high affinity and specificity toward linear dodecapeptide antigen, PMab-1 can be used to detect and purify tagged proteins in various assay formats. In the near future, we will compare the MAP tag to several other affinity tag systems against many targets and determine the types of applications most suitable for the MAP tag system.

## References

[1] Smith DB, and Johnson KS: Single-step purification of polypeptides expressed in *Escherichia coli* as fusions with glutathione S-transferase. Gene 1988;67:31–40 3047011 10.1016/0378-1119(88)90005-4

[2] Diguan C, Li P, Riggs PD, and Inouye H: Vectors that facilitate the expression and purification of foreign peptides in *Escherichia coli* by fusion to maltose-binding protein. Gene 1988;67:21–30 2843437 10.1016/0378-1119(88)90004-2

[3] Hochuli E, Bannwarth W, Dobeli H, Gentz R, and Stuber D: Genetic approach to facilitate purification of recombinant proteins with a novel metal chelate adsorbent. Biotechnology 1988;6:1321–1325

[4] Hopp TP, Prickett KS, Price VL, Libby RT, March CJ, Cerretti DP, Urdal DL, and Conlon PJ: A short polypeptide marker sequence useful for recombinant protein identification and purification. Biotechnology 1988;6:1204–1210

[5] Tabata S, Nampo M, Mihara E, Tamura-Kawakami K, Fujii I, and Takagi J: A rapid screening method for cell lines producing singly-tagged recombinant proteins using the “TARGET tag” system. J Proteomics 2010;73:1777–1785 20566373 10.1016/j.jprot.2010.05.012

[6] Fujii Y, Kaneko M, Neyazaki M, Nogi T, Kato Y, and Takagi J: PA tag: A versatile protein tagging system using a super high affinity antibody against a dodecapeptide derived from human podoplanin. Protein Expr Purif 2014;95:240–247 24480187 10.1016/j.pep.2014.01.009

[7] Fujii Y, Matsunaga Y, Arimori T, Kitago Y, Ogasawara S, Kaneko MK, Kato Y, and Takagi J: Tailored placement of a turn-forming PA tag into the structured domain of a protein to probe its conformational state. J Cell Sci 2016;129:1512–1522 26872787 10.1242/jcs.176685

[8] Yano T, Takeda H, Uematsu A, Yamanaka S, Nomura S, Nemoto K, Iwasaki T, Takahashi H, and Sawasaki T: AGIA tag system based on a high affinity rabbit monoclonal antibody against human dopamine receptor D1 for protein analysis. PLoS One 2016;11:e0156716 27271343 10.1371/journal.pone.0156716 PMC4894603

[9] Field J, Nikawa J, Broek D, Macdonald B, Rodgers L, Wilson IA, Lerner RA, and Wigler M: Purification of a ras-responsive adenylyl cyclase complex from *Saccharomyces Cerevisiae* by use of an epitope addition method. Mol Cell Biol 1988;8:2159–2165 2455217 10.1128/mcb.8.5.2159-2165.1988 PMC363397

[10] Evan GI, Lewis GK, Ramsay G, and Bishop JM: Isolation of monoclonal antibodies specific for human c-myc proto-oncogene product. Mol Cell Biol 1985;5:3610–3616 3915782 10.1128/mcb.5.12.3610-3616.1985 PMC369192

[11] Lichty JJ, Malecki JL, Agnew HD, Michelson-Horowitz DJ, and Tan S: Comparison of affinity tags for protein purification. Protein Expr Purif 2005;41:98–105 15802226 10.1016/j.pep.2005.01.019

[12] Kato Y, Fujita N, Kunita A, Sato S, Kaneko M, Osawa M, and Tsuruo T: Molecular identification of Aggrus/T1alpha as a platelet aggregation-inducing factor expressed in colorectal tumors. J Biol Chem 2003;278:51599–51605 14522983 10.1074/jbc.M309935200

[13] Sasaki F, Okuno T, Saeki K, Min L, Onohara N, Kato H, Shimizu T, and Yokomizo T: A high-affinity monoclonal antibody against the FLAG tag useful for G-protein-coupled receptor study. Anal Biochem 2012;425:157–165 22465329 10.1016/j.ab.2012.03.014

[14] Einhauer A, and Jungbauer A: The FLAG peptide, a versatile fusion tag for the purification of recombinant proteins. J Biochem Biophys Methods 2001;49:455–465 11694294 10.1016/s0165-022x(01)00213-5

[15] Kaji C, Tsujimoto Y, Kato Kaneko M, Kato Y, and Sawa Y: Immunohistochemical examination of novel rat monoclonal antibodies against mouse and human podoplanin. Acta Histochem Cytochem 2012;45:227–237 23012488 10.1267/ahc.12008 PMC3445762

[16] Kato Y, Kaneko MK, Kunita A, Ito H, Kameyama A, Ogasawara S, Matsuura N, Hasegawa Y, Suzuki-Inoue K, Inoue O, Ozaki Y, and Narimatsu H: Molecular analysis of the pathophysiological binding of the platelet aggregation-inducing factor podoplanin to the C-type lectin-like receptor CLEC-2. Cancer Sci 2008;99:54–61 17944973 10.1111/j.1349-7006.2007.00634.x PMC11159596

[17] Kaneko MK, Kato Y, Kitano T, and Osawa M: Conservation of a platelet activating domain of Aggrus/podoplanin as a platelet aggregation-inducing factor. Gene 2006;378:52–57 16766141 10.1016/j.gene.2006.04.023

[18] Oki H, Honma R, Ogasawara S, Fujii Y, Liu X, Takagi M, Kaneko MK, and Kato Y: Development of sensitive monoclonal antibody PMab-2 against rat podoplanin. Monoclon Antib Immunodiagn Immunother 2015;34:396–403 26683179 10.1089/mab.2015.0041

[19] Picketts DJ, Higgs DR, Bachoo S, Blake DJ, Quarrell OW, and Gibbons RJ: ATRX encodes a novel member of the SNF2 family of protein: Mutations point to a common mechanism underlying the ATR-X syndrome. Hum Mol Genet 1996;5:1899–1907 8968741 10.1093/hmg/5.12.1899

[20] Ogasawara S, Kaneko MK, and Kato Y: LpMab-19 Recognizes sialylated O-glycan on Thr76 of human podoplanin. Monoclon Antib Immunodiagn Immunother. 2016;35:245–253 10.1089/mab.2016.0031 27564251

[21] Ogasawara S, Kaneko MK, Tsujimoto Y, Liu X, and Kato Y: Multi-specific monoclonal antibody MsMab-2 recognizes IDH1-R132L and IDH2-R172M mutations. Monoclon Antib Immunodiagn Immunother 2013;32:377–381 24328739 10.1089/mab.2013.0050

[22] Kato Y, Ogasawara S, Oki H, Honma R, Takagi M, Fujii Y, Nakamura T, Saidoh N, Kanno H, Umetsu M, Kamata S, Kubo H, Yamada M, Sawa Y, Morita K, Harada H, Suzuki H, and Kaneko MK: Novel monoclonal antibody LpMab-17 developed by CasMab technology distinguishes human podoplanin from monkey podoplanin. Monoclon Antib Immunodiagn Immunother 2016;35:109–116 26937552 10.1089/mab.2015.0077

[23] Suzuki H, Kato Y, Kaneko MK, Okita Y, Narimatsu H, and Kato M: Induction of podoplanin by transforming growth factor-beta in human fibrosarcoma. FEBS Lett 2008;582:341–345 18158922 10.1016/j.febslet.2007.12.028

